# SARS-CoV-2 Omicron variants BA.1 and BA.2 both show similarly reduced disease severity of COVID-19 compared to Delta, Germany, 2021 to 2022

**DOI:** 10.2807/1560-7917.ES.2022.27.22.2200396

**Published:** 2022-06-02

**Authors:** Claudia Sievers, Benedikt Zacher, Alexander Ullrich, Matthew Huska, Stephan Fuchs, Silke Buda, Walter Haas, Michaela Diercke, Matthias an der Heiden, Stefan Kröger

**Affiliations:** 1 Department of Infectious Disease Epidemiology, Robert Koch Institute, Berlin, Germany; 2 Postgraduate Training for Applied Epidemiology (PAE), Robert Koch Institute, Berlin, Germany; 3 European Programme for Intervention Epidemiology Training (EPIET), European Centre for Disease Prevention and Control (ECDC), Stockholm, Sweden; 4 These authors contributed equally to this work and share first authorship; 5 Department of Genome Sequencing and Genomic Epidemiology, Robert Koch Institute, Berlin, Germany

**Keywords:** SARS-CoV-2, COVID-19, Omicron, BA.1, BA.2, Delta, VOC

## Abstract

German national surveillance data analysis shows that hospitalisation odds associated with Omicron lineage BA.1 or BA.2 infections are up to 80% lower than with Delta infection, primarily in ≥ 35-year-olds. Hospitalised vaccinated Omicron cases’ proportions (2.3% for both lineages) seemed lower than those of the unvaccinated (4.4% for both lineages). Independent of vaccination status, the hospitalisation frequency among cases with Delta seemed nearly threefold higher (8.3%) than with Omicron (3.0% for both lineages), suggesting that Omicron inherently causes less severe disease.

Since week 46 2021, when it was first reported in South Africa, the severe acute respiratory syndrome coronavirus 2 (SARS-CoV-2) variant of concern (VOC) Omicron (Phylogenetic Assignment of Named Global Outbreak (Pango) lineage: B.1.1.529) sub-lineage BA.1 started circulating in Germany [1]. Subsequently, in week 51 2021 the sub-lineage BA.2 started to appear in the country. While BA.1 transmissibility is increased compared to that of the Delta VOC (Pango lineage: B.1.617.2) [2,3], BA.2 appears to have a higher effective reproduction number than BA.1 [4] and has become the dominant variant in Germany in week 08 2022 [5]. Early reports have suggested that BA.1 infection is less likely to result in severe disease than Delta infection [6-10], but little is known on the chances of severe disease associated with BA.2 infection [11,12].

Based on variant typing using whole genome sequencing (WGS) and epidemiological data from the German national surveillance database, this study aimed to estimate the odds ratios for severe disease progression (hospitalisation, admission to intensive care unit (ICU) or death) associated with BA.1 and BA.2 infections compared with Delta infection.

## Study population

Coronavirus disease (COVID-19) cases with a WGS-confirmed BA.1, BA.2 or Delta SARS-CoV-2 variant infection (including their respective sub-lineages), notified to the German national surveillance system between 1 November 2021 and 15 April 2022 were analysed as a retrospective cohort.

**Figure 1:**
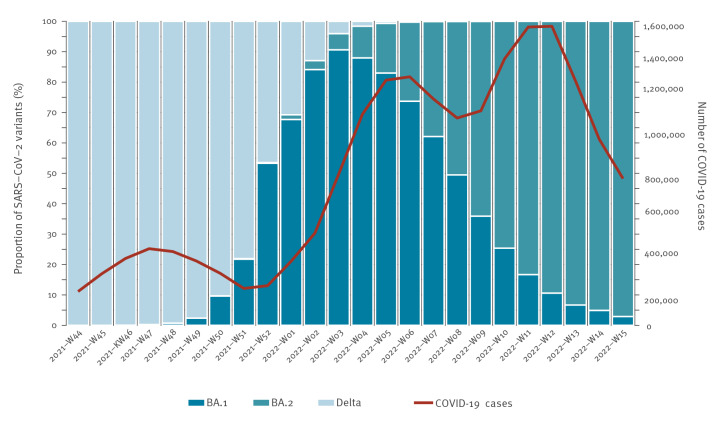
SARS-CoV-2 variants Delta, BA.1 and BA.2’s weekly distribution^a^ and number of notified COVID-19 cases, Germany, 1 November 2021–15 April 2022 (n = 238,107)

COVID-19: coronavirus disease; SARS-CoV-2: severe acute respiratory coronavirus 2.

^a^ The distribution is according to the whole genome sequencing result of samples randomly selected from the pool of all SARS-CoV-2 positive samples.

Data were extracted on 29 April 2022, allowing for a reporting delay of 14 days of an eventual hospitalisation. A subset of cases with a notification date between 1 November 2021 and 1 April 2022 was used to analyse deaths, allowing a follow-up time of 28 days. Figure 1 shows the distribution of cases by variant and the sharp increase in total COVID-19 cases in Germany beginning in week 02 2022.

For the hospitalisation dataset, cases were excluded if hospitalisation, vaccination status (Supplementary Figure S1) or information for any adjustment variables was missing. Cases were also excluded if they had a prior SARS-CoV-2 infection reported. Of initially 238,107 COVID-19 cases linked to WGS, 47,160 cases were included in the analysis. The characteristics of the hospitalised study population are listed in Table 1. The selection of a subset of cases suitable for investigating odds of death relative to different SARS-CoV-2-variant infections is described in Supplementary Figure S1. The demographical features, vaccination status and outcome of cases in this subset are displayed according to each variant infection in Supplementary Table S1. 

**Table 1:** Characteristics of COVID-19 cases included in the retrospective cohort to study odds of hospitalisation and severe disease, according to infection with Delta, Omicron BA.1 and BA.2 SARS-CoV-2 variants, Germany, 1 November 2021–15 April 2022 (n = 47,160)

Characteristic	Delta (B.1.617.2) (incl. sub-lineages)		Omicron (B.1.1.529)				Total	
			BA.1 (incl. sub-lineages)		BA.2 (incl. sub-lineages)			
	Number	%	Number	%	Number	%	Number	%
**Total**	24,530	52.0	15,770	33.4	6,860	14.5	47,160	100.0
**Sex^a^**								
Female	12,401	50.6	8,137	51.6	3,695	53.9	24,233	51.4
Male	12,129	49.4	7,633	48.4	3,165	46.1	22,927	48.6
**Age group (years)**								
0–4	673	2.7	483	3.1	185	2.7	1,341	2.8
5–14	4,557	18.6	2,255	14.3	792	11.5	7,604	16.1
15–34	6,190	25.2	5,722	36.3	2,434	35.5	14,346	30.4
35–59	8,807	35.9	5,539	35.1	2,406	35.1	16,752	35.5
60–79	3,113	12.7	1,238	7.9	728	10.6	5,079	10.8
≥ 80	1,190	4.9	533	3.4	315	4.6	2,038	4.3
**Hospitalisation**								
Yes	2,043	8.3	472	3.0	203	3.0	2,718	5.8
No	22,487	91.7	15,298	97.0	6,657	97.0	44,442	94.2
**ICU-treatment^b^**								
Yes	403	1.7	43	0.3	19	0.3	465	1.0
No	24,013	98.3	15,691	99.7	6,827	99.7	46,531	99.0
**Vaccination status**								
Unvaccinated	14,048	57.3	5,330	33.8	1,897	27.7	21,275	45.1
Vaccinated	9,950	40.6	6,269	39.8	1,554	22.7	17,773	37.7
Booster vaccinated	532	2.2	4,171	26.4	3,409	49.7	8,112	17.2

COVID-19: coronavirus disease; ICU: intensive care unit; SARS-CoV-2: severe acute respiratory coronavirus 2.

^a^ Cases with sex recorded as ‘diverse’ were excluded due to the low sample size (n = 20) as were cases with sex unknown (n = 486).

^b^ 114 cases with Delta, 36 with BA.1 and 14 with BA.2 infection had information missing as to whether they were treated or not in ICU.

Due to the high number of missing values, in particular for hospitalisation or vaccination status, 191,947 cases (80% of 238,107 cases linked to WGS data) were omitted from the final study population for hospitalisation (Supplementary Figure S1). A comparison of the adjustment variables shows a similar distribution for included cases as for the overall population (Supplementary Table S4). However, the distribution of BA.1, BA.2 and Delta VOCs was different between the two populations.

## Hospitalisation

The percentage of hospitalisations following an infection with Delta (8.3%) seemed almost threefold higher than with BA.1 and BA.2 (both 3.0%, Table 1). This effect was particularly evident in the age groups 35 years and above, where the proportion of hospitalisations appeared to be nearly three times higher for Delta infections (13.6%; 1,811/13,310) than for BA.1 (5.0%; 362/7,310) or BA.2 (4.8%; 165/3,449) infections. In contrast, for children < 15 years old there did not seem to be any difference between Delta (1.5%; 77/5,230) and BA.1 (1.5%; 40/2,738) or BA.2 (1.4%; 14/977) infections in terms of frequency of hospitalisation (Table 2).

**Table 2:** Odds ratios of hospitalisation, ICU admission and death after infection with SARS-CoV-2 Omicron BA.1 or BA.2 variants compared with Delta, overall and according to age group or vaccination status, Germany, 1 November 2021–15 April 2022 (n = 47,160)

Outcome	Delta		Omicron BA.1		Omicron BA.2		BA.1 vs Delta	BA.2 vs Delta
	n/N	%	n/N	%	n/N	%	Adjusted OR^a,b^ (95% CI)	Adjusted OR^a,b^ (95% CI)
**Hospitalisation**	2,043/24,530	8.3	472/15,770	3.0	203/6,860	3.0	0.35 (0.29–0.43)***	0.30 (0.22–0.40)***
**ICU admission^c^**	403/24,416	1.7	43/15,734	0.3	19/6,846	0.3	0.20 (0.12–0.32)***	0.17 (0.07–0.39)***
**Deaths^d^**	545/31,720	1.7	96/20,818	0.5	26/7,143	0.4	0.38 (0.25–0.58)***	0.16 (0.08–0.30)***
**Subgroup analysis for hospitalisation^b^**								
Age group (years)								
0–4	36/673	5.3	21/483	4.3	10/185	5.4	0.58 (0.25–1.33)	0.74 (0.24–2.25)
5–14	41/4,557	0.9	19/2,255	0.8	4/792	0.5	0.72 (0.31–1.64)	0.43 (0.09–2.00)
15–34	155/6,190	2.5	70/5,722	1.2	24/2,434	1.0	0.50 (0.31–0.80)***	0.43 (0.21–0.91)*
35–59	556/8,807	6.3	86/5,539	1.6	31/2,406	1.3	0.23 (0.15–0.35)***	0.20 (0.10–0.38)***
60–79	685/3,113	22.0	128/1,238	10.3	55/728	7.6	0.38 (0.25–0.56)***	0.27 (0.15–0.49)***
≥ 80	570/1,190	47.9	148/533	27.8	79/315	25.1	0.33 (0.21–0.52)***	0.29 (0.16–0.53)***
Vaccination status								
Unvaccinated	1,275/14,048	9.1	228/5,330	4.3	90/1,897	4.7	0.34 (0.25–0.47)***	0.28 (0.18–0.45)***
Vaccinated	691/9,950	6.9	126/6,269	2.0	32/1,554	2.1	0.39 (0.28–0.55)***	0.32 (0.17–0.59)***
Booster vaccinated	77/532	14.5	118/4,171	2.8	81/3,409	2.4	0.30 (0.19–0.50)***	0.27 (0.15–0.49)***

CI: confidence interval; COVID-19: coronavirus disease; ICU: intensive care unit; NA: not applicable; OR: odds ratio; SARS-CoV-2: severe acute respiratory coronavirus 2.

**^a^** For hospitalisation overall, ICU admission and death, the ORs were adjusted for age group, vaccination status, sex, federal state of notifying health authority and week of notification.

**^b^** For the hospitalisation sub-analyses the ORs were adjusted for sex, federal state of notifying health authority, week of notification and vaccination status or age group, respectively

^c^ The study population contains 164 less cases than that for hospitalisation due to missing values in the variable ICU admission.

^d^ This study population consists of COVID-19 cases notified between 1 November 2021 and 1 April 2022 (n = 59,681 cases), see Supplementary Figure S1 and Table S1.

*p value: p < 0.05: *, p < 0.01: **, p < 0.001: ***

**Figure 2:**
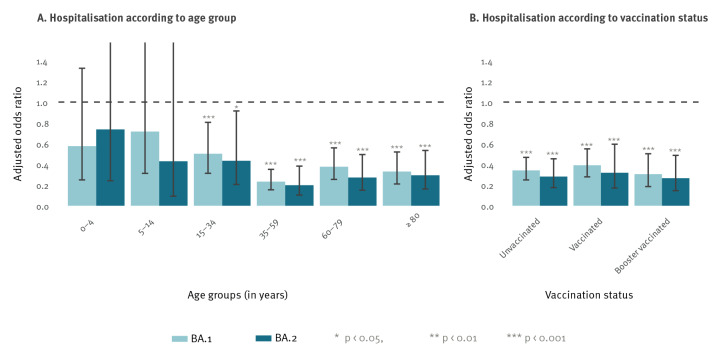
Odds ratios of hospitalisation after infection with SARS-CoV-2 Omicron BA.1 or BA.2 variants compared with Delta according to age group or vaccination status, Germany, 1 November 2021–15 April 2022 (n = 47,160)

CI: confidence interval; SARS-CoV-2: severe acute coronavirus 2.

To estimate the odds for a variant-specific hospitalisation, a multivariable logistic regression model was used, adjusting for age group, vaccination status, sex, federal state of notifying health authority and calendar week of notification. Similar reduced adjusted odds ratios (adjOR) of hospitalisation were obtained when considering BA.1 and BA.2 Omicron variant infections compared with a Delta variant infection (adjOR BA.1: 0.35; 95% CI: 0.29–0.43 and adjOR BA.2: 0.30; 95% CI: 0.22–0.40) (Table 2). Stratification for age showed, that younger age groups (0–14-year-olds) had no significant difference in hospitalisation depending on the variant, but a strong effect was observed in age groups 35 years and above with the odds of hospitalisation reduced up to 80% for BA.1 and BA.2 (Figure 2 and Table 2). When stratifying by vaccination status, the proportions of hospitalisations for (booster-) vaccinated cases compared with unvaccinated cases appeared to be reduced (for BA.1: 2.3% hospitalisations (244/10,440) among (booster-) vaccinated cases vs 4.3% among unvaccinated; for BA.2: 2.3% (113/4,963) hospitalisations among (booster-) vaccinated cases vs 4.7% among unvaccinated; for Delta: 7.3% (768/10,482) hospitalisations among (booster-) vaccinated cases vs 9.1% among unvaccinated cases). Moreover, for all vaccination statuses, a significant decrease was observed for the odds of being hospitalised after BA.1 or BA.2 infection compared with Delta (Figure 2 and Table 2). Additionally, when the timing of the last vaccination (< 90 days, 90 to 180 days, > 180 days) was considered (Supplementary Figure S2), ORs for hospitalisations remained similar to those in Figure 2. Stratification by age and vaccination status (Supplementary Table S5) showed comparable results, but with wider confidence intervals and not for all age groups because of small group sizes for (booster-) vaccinated children and (booster-) vaccinated Delta cases.

Error bars show the 95% CIs, cut at 1.6 for age groups (in years), to increase comparability. The full 95% CIs are shown in Table 2; p-value: p < 0.05: *, p < 0.01: **, p < 0.001: ***

## Intensive care unit admission

Using the same covariates in the logistic regression model as for hospitalisation, the odds of a COVID-19 case to be admitted to ICU were estimated to be reduced by more than 80% for both BA.1 and BA.2 compared with Delta (adjOR BA.1: 0.20 (95% CI: 0.12–0.32); adjOR BA.2: 0.17 (95% CI: 0.07–0.39)).

## Reported deaths

In the total study population selected for the analysis of deaths as outcome (n = 59,681), 667 deaths (1.1%) were recorded, of which 96 (0.5%) occurred among 20,818 cases infected with BA.1 and 26 (0.4%) among 7,143 with BA.2. Using the same covariates in the logistic regression model as for hospitalisation, the analysis showed reduced odds for dying upon an infection with BA.1 or BA.2 compared with Delta (adjOR BA.1: 0.38 (95% CI: 0.25–0.58); adjOR BA.2: 0.16 (95% CI: 0.08–0.3)) (Table 2). Details on the study population are depicted in Supplementary Table S1.

## Discussion

In this study, we found that the odds of hospitalisation following a BA.1 or BA.2 Omicron variant infection was up to 80% lower than following a Delta variant infection, particularly in adults ≥ 35 years old. Both BA.1 and BA.2 had a similar effect on hospitalisation or ICU admission, suggesting that despite reports of increased transmissibility for BA.2 [4], this variant does not differ from BA.1 in pathogenicity. In support of our results, no clinical differences were found between individuals infected with BA.1 and BA.2 in Denmark [12], a country with similar demographics as Germany and a high vaccination coverage in older people – the vaccination rate among the age group above 60 years in Germany is > 90% [13]. In a different setting presented by South Africa, which has a younger population (91% of the population is <60 years old there [14], as opposed to 71% in Germany [15]) and where immunity to SARS-CoV-2 is more owed to previous infections, the clinical profile of illness due to BA.1 and BA.2 was also more or less the same [11]. The overall odds ratio of 0.33 for hospitalisation for BA.1 in our study was moreover similar to hazard, risk or odds ratios reported by other authors, ranging between 0.2 and 0.65 [6,8-10,16].

Our analysis indicates that the reduction in disease severity observed with BA.1 and BA.2 variant infections compared to Delta might be age dependent. The reduction compared with Delta was evident in older age groups but not in children (0–14 years-olds), who generally have a low risk for COVID-19 related-hospitalisation [17]. As previously observed within the German surveillance data on COVID-19 and elsewhere, the strongest association with hospitalisation is age, especially in age groups above 60 years [18]. Unlike in South Africa, we did not observe an increase in hospitalisations in children under 5 years of age with Omicron infection. However, the lack of reduction in hospitalisations in South Africa for 5−12-year-olds infected with Omicron relative to Delta is consistent with our results and what has been observed in England [6,8]. Our data might, however, be biased towards overestimation of the chance of hospitalisation for children, as we did not differentiate for ‘hospitalisation because of COVID-19’ and ‘hospitalisation with COVID-19’. Beginning with week 03 2022, COVID-19 incidences were highest among children aged 5–14 years [13], coinciding with the peak of BA.1 cases. Thus, it is likely that children have been increasingly hospitalised ‘with’ COVID-19, resulting in an underestimation of a reduction in hospitalisation related to Omicron infection compared to Delta infection.

(Booster-) vaccination reduces the proportion of hospitalised cases and for all groups (unvaccinated and (booster-) vaccinated cases) the chance of being hospitalised is reduced for BA.1 and BA.2 cases compared with Delta cases. This seems independent of how long ago the last vaccination took place, suggesting that both Omicron variants show an intrinsic reduction in their pathogenicity.

To minimise underestimation on the risk of hospitalisation due to the effect of prior infections, which have been shown to have a protective effect [8], we excluded cases notified as re-infected from our analysis. Although, this does not correct for the inclusion of cases with unknown/unnotified prior infection, in Germany, seroprevalence and under-reporting of cases were low until August 2021 [19]. Underestimation of BA.1 severity due to inclusion of unknown prior infections with Delta is therefore estimated to be small. With increased incidence of COVID-19 cases starting in the beginning of 2022 and strained testing capacity, under-ascertainment of BA.1 cases may have led to underestimation of hospitalisation due to BA.2 infection. However, all vaccination groups are equally affected by under-reporting, and other reports have shown minimal effects of under-reporting of prior infections on the risk of hospitalisation [8].

With the strong increase in COVID-19 cases, health authorities increasingly prioritised the data entry on selected variables, especially hospitalisation, depending on the federal state. Since July 2021, additional notification requirements for hospitalised patients have been implemented and for those cases, vaccination status is systematically collected, while cases with only laboratory confirmation require active investigation of the vaccination status by local health authorities. Thus, hospitalised cases more often have complete information of the vaccination status, leading the current study population to seemingly have more hospitalised cases over time and in certain federal states (since cases with unknown vaccination status are excluded, Supplementary Table S2). While the distribution of cases for the descriptive variables was similar in the final study population compared with the overall population, the differences for the proportion of Delta, BA.1 and BA.2 cases could be an indirect result of the observed reduced severity for Omicron. As the final study population is biased on the inclusion of hospitalised cases (see above) and Omicron leads to a reduction in hospitalisation, its proportions are lower in the final study population.

Additionally, to assess the bias due to more complete data entry at the beginning of the occurrence of a new VOC, here BA.1 in November 2021, we analysed a WGS-dataset of specimens which were randomly selected for sequencing from the pool of all SARS-CoV-2 positive samples. All results were consistent with those from the complete dataset (Supplementary Table S3).

## Conclusion

Overall, people infected with Omicron variants BA.1 and BA.2 are similarly less likely to progress to hospitalisation compared with those infected with Delta. This effect is particularly evident in adults (≥ 35 years old) as well as in both unvaccinated and (booster-) vaccinated cases (for all age groups).

## Supplementary Data

378000 Supplementary Material
