# Supplementary material for: SARS-CoV-2 Omicron variants BA.1 and BA.2 both show similarly reduced disease severity of COVID-19 compared to Delta, Germany, 2021 to 2022
Source: Euro Surveill. 2022 Jun 2;27(22):2200396. doi: 10.2807/1560-7917.ES.2022.27.22.2200396 (PMC9164675; doi:10.2807/1560-7917.ES.2022.27.22.2200396)
Supplement: Supplementary Material [file 2200396_SupplementaryMaterial.pdf]

Supplementary material: SARS-CoV-2 Omicron variants BA.1 and BA.2 both show similarly reduced disease severity of COVID-19 compared to Delta, Germany, 2021 to 2022

"This supplementary material is hosted by Eurosurveillance as supporting information alongside the article "SARS-CoV-2 Omicron variants BA.1 and BA.2 both show similarly reduced disease severity of COVID-19 compared to Delta, Germany, 2021 to 2022", on behalf of the authors, who remain responsible for the accuracy and appropriateness of the content. The same standards for ethics, copyright, attributions and permissions as for the article apply. Supplements are not edited by Eurosurveillance and the journal is not responsible for the maintenance of any links or email addresses provided therein."

Additional information on the study population:

Cases confirmed by RNA detection or virus isolation fulfill the reference definition and were included in the present analysis.

Vaccination status

The vaccination status unvaccinated, fully vaccinated or having received a booster dose was defined as follows: cases were considered as i) unvaccinated if they were reported as not having been vaccinated, as ii) fully vaccinated if they received two vaccination doses of Comirnaty (BioNTech/Pfizer), Spikevax (Moderna) or Vaxzevria (AstraZeneca) or combinations thereof or one dose of Janssen at least 14 days before the date of onset or alternatively the notification date or if they received an additional vaccine dose less than 7 days before the date of onset or alternatively the notification date, or as iii) having received a booster dose with at least 3 doses of a COVID-19 vaccine or specifically 2 doses if the first was with Janssen, at least 7 days before the date of onset or alternatively the notification date. Cases which could not be assigned to the three definitions as described, were excluded from the analysis.

Additionally, vaccinated cases were divided into three subgroups that included duration since last vaccination (<90 days, 90 to 180 days, >180 days).

Prior infections

Cases registered as having had a prior infection by the local health authorities were excluded from the analysis.

Reported deaths

The data sub-set for the analysis of an association between VOC and death (notification date between November 1st, 2021 and April 15th, 2022) included complete variables for vaccination status, sex and age. As no adjustment was done for hospitalisation or ICU admission, cases with missing information on these were included in the final study population.

Whole genome sequencing

With the date of data closures (April 29th, 2022) 468,515 WGS of SARS-CoV-2 variants had been submitted to the German electronic sequencing hub (DESH) for the study period. Out of these, 227,397 were submitted as random samples, chosen by using a random sampling strategy from all SARS-CoV-2 positive samples, to be sequenced. The SARS-CoV-2 lineages were assigned at the Robert Koch-Institute on the basis of the most recent pangolin version (pangolin 4.0.6, pangoLEARN 2022-03-22, scorpio 0.3.17, pangolin-data 1.6 and pango constellations 0.1.8. https://cov-lineages.org/resources/pangolin.html). Of all uploaded sequences that fulfilled the quality criteria 238,107 sequences could be linked to the national SARS-CoV-2 surveillance data (51%). Using only the random sample, 114,831 sequences could be linked.

Statistical analysis

Hospitalisation, admission to an intensive care unit and death were used as outcomes, in order to compare disease severity between SARS-CoV-2 variants.

Plotting the following directed acyclic graph (http://dagitty.net/dags.html?id=hgdqou) identified this adjustment set for the outcome "recorded hospitalisation":

- Age group: 0-4, 5-14, 15-34, 35-59, 40-59, 60-79 and >79 years.
- Vaccination status: unvaccinated, fully vaccinated or having received a booster dose
- Federal state of notifying health authority.
- Week of case notification: calendar week 44 in 2021 until calendar week 16 in 2022

We additionally included the variable gender (male and female) in the multivariable logistic regression models to estimate adjusted odds ratios of SARS-CoV-2 variants. There were 20 cases with gender diverse, which were excluded from the analysis due to the low number.
The models used for the three different outcomes are explained in the following sections. All analyses were performed using the statistical programming language R (version 3.6.1. and version 4.1.2). Null-hypotheses were tested using the multcomp package.

Hospitalisation

First, a model was considered, which estimated an effect of the SARS-CoV-2 variant on the outcome hospitalisation, which was independent of all other considered variables (i.e. no interaction terms). To this end, we included all variables listed above in the model. All likelihood ratio tests (LR-test) of one iteration of backward selection – where one variable was removed at a time – were significant and thus, all variables were kept in the final model. In order to get age- and vaccination status-dependent estimates, we further included interaction terms.

Admission to an intensive care unit and death

Model fitting and selection was done as with outcome hospitalisation. Likewise, the complete model was significantly better than all other models, where one variable was removed. Since the number of cases admitted to an intensive care unit and the number of deaths was low for BA.1 and BA.2 (see Table 2), age- and vaccination status-dependent estimates were not computed.

Figure S1: Flow chart of the selection of cases to analyse the association of SARS-Cov-2 variants Delta, BA.1 and BA.2 on hospitalisation and deaths Germany, 1 November 2021 – 15 April 2022

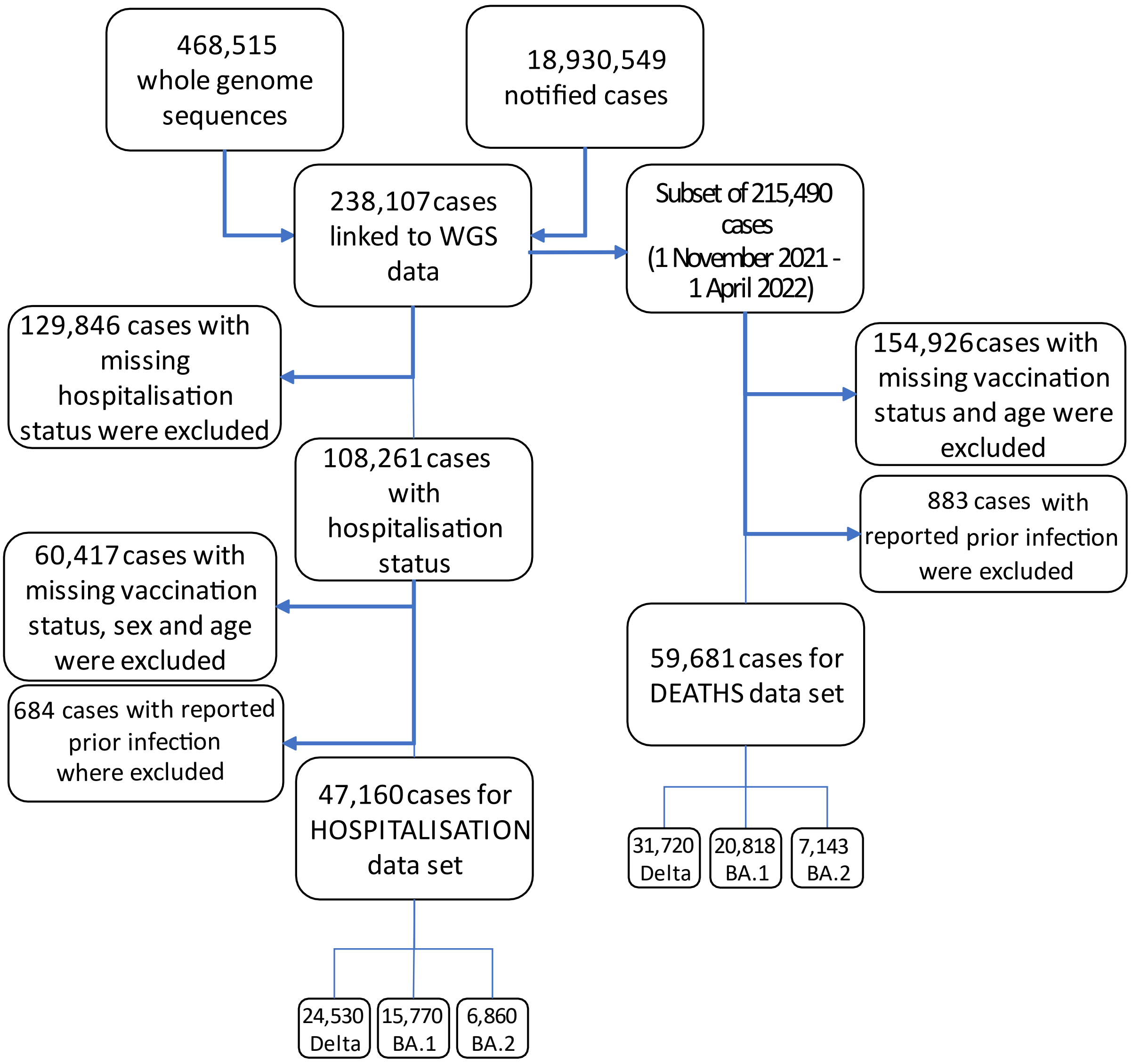

Table S1: Characteristics of SARS-CoV-2 cases in the retrospective cohort study on deaths, by variants Delta, BA.1 and BA.2 for characteristics variables, Germany, 1 November 2021 – 1 April 2022 (n=59,681).

Characteristic	Delta Variant (B.1.617.2 incl. AY.* lineages)		Omicron -BA.1 variant (incl. sub-lineages)		Omicron- BA.2 variant (incl. sub-lineages)		Total	
**Sub-analysis for deaths:**	N	%	N	%	N	%	N	%
**Total**	31720	53.1	20818	34.9	7143	12	59681	100
**Sex**								
Female	16121	50.8	10809	51.9	3846	53.8	30776	51.6
Male	15599	49.2	10009	48.1	3297	46.2	28905	48.4
**Age groups (years)**								
0 - 4	855	2.7	663	3.2	218	3.1	1736	2.9
5 - 14	5849	18.4	3001	14.4	919	12.9	9769	16.4
15 - 34	8041	25.3	7541	36.2	2385	33.4	17967	30.1
35 - 59	11621	36.6	7226	34.7	2557	35.8	21404	35.9
60 - 79	3941	12.4	1676	8.1	676	9.5	6293	10.5
≥80	1413	4.5	711	3.4	388	5.4	2512	4.2
**Vaccination status**								
Unvaccinated	18473	58.2	7216	34.7	2173	30.4	27862	46.7
Vaccinated	12582	39.7	8246	39.6	1721	24.1	22549	37.8
Booster vaccinated	665	2.1	5356	25.7	3249	45.5	9270	15.5
**Deaths**								
Yes	545	1.7	96	0.5	26	0.4	667	1.1
No	31175	98.3	20722	99.5	7117	99.6	59014	98.9

Table S2: Odds Ratio for covariates sex, age group, vaccination status, federal state of notifying health authority and week of notification on hospitalisation, adjusted for the respective other variables and VOC (Delta, BA.1 and BA.2), Germany, 1 November 2021 – 15 April 2022 (n=47,160).

**Categories**	**n/N**	**%**	**adj. OR (95% CI)**
**Sex**			
Female	1273/24233	5.3	Reference
Male	1445/22927	6.3	1.48 (1.35 - 1.61) ***
**Age group (years)**			
0 - 4	67/1341	5.0	Reference
5 - 14	64/7604	0.8	0.16 (0.11 - 0.23) ***
15 - 34	249/14346	1.7	0.72 (0.55 - 0.97) *
35 - 59	673/16752	4.0	1.70 (1.31 - 2.23) ***
60 - 79	868/5079	17.1	9.33 (7.18 - 12.31) ***
≥80	797/2038	39.1	36.77 (27.95 - 49.06) ***
**Vaccination status**			
Unvaccinated	1593/21275	7.5	Reference
Vaccinated	849/17773	4.8	0.31 (0.28 - 0.35) ***
Booster vaccinated	276/8112	3.4	0.19 (0.16 - 0.23) ***

Federal state of health authority	n/N	%	adj. OR (95% CI)
1	729/6178	11.8	Reference
2	427/13492	3.2	0.28 (0.25 - 0.33) ***
3	91/1767	5.1	0.51 (0.39 - 0.66) ***
4	88/819	10.7	0.89 (0.68 - 1.16)
5	8/102	7.8	1.35 (0.55 - 2.91)
6	82/1123	7.3	0.80 (0.60 - 1.05)
7	163/2156	7.6	0.73 (0.60 - 0.89) **
8	82/812	10.1	0.81 (0.61 - 1.07)
9	61/1929	3.2	0.29 (0.21 - 0.38) ***
10	390/10962	3.6	0.33 (0.29 - 0.39) ***
11	205/2499	8.2	0.70 (0.58 - 0.84) ***
12	21/613	3.4	0.26 (0.16 - 0.41) ***
13	245/1088	22.5	1.48 (1.22 - 1.81) ***
14	50/1582	3.2	0.16 (0.11 - 0.22) ***
15	24/1047	2.3	0.21 (0.13 - 0.32) ***
16	52/991	5.2	0.35 (0.25 - 0.47) ***
**Week of notification**			
2021W44	244/3242	7.5	Reference
2021W45	202/2716	7.4	1.21 (0.98 - 1.51)
2021W46	227/2835	8.0	1.28 (1.04 - 1.59) *
2021W47	252/3352	7.5	1.40 (1.13 - 1.72) **
2021W48	249/3337	7.5	1.38 (1.12 - 1.70) **
2021W49	249/3211	7.8	1.38 (1.12 - 1.71) **
2021W50	232/3274	7.1	1.36 (1.10 - 1.69) **
2021W51	170/2330	7.3	1.75 (1.37 - 2.21) ***
2021W52	154/3063	5.0	1.80 (1.40 - 2.32) ***
2022W1	136/2974	4.6	1.99 (1.52 - 2.61) ***
2022W2	89/2360	3.8	2.21 (1.61 - 3.03) ***
2022W3	66/2053	3.2	2.35 (1.64 - 3.33) ***
2022W4	51/1468	3.5	2.53 (1.70 - 3.74) ***
2022W5	49/1238	4.0	2.70 (1.79 - 4.03) ***
2022W6	67/1641	4.1	2.74 (1.87 – 4.00) ***
2022W7	54/1290	4.2	3.18 (2.11 - 4.77) ***
2022W8	54/1028	5.3	4.16 (2.71 - 6.31) ***
2022W9	48/1021	4.7	4.80 (3.06 - 7.43) ***
2022W10	40/1256	3.2	3.84 (2.40 - 6.05) ***
2022W11	21/865	2.4	2.29 (1.27 - 3.99) **
2022W12	16/669	2.4	2.58 (1.33 - 4.74) **
2022W13	20/733	2.7	2.46 (1.31 - 4.43) **
2022W14	15/684	2.2	2.59 (1.31 - 4.85) **
2022W15	13/520	2.5	2.93 (1.39 - 5.77) **

Adj. OR: adjusted odds ratio CI: confidence interval;

Table S3: Odds Ratio of hospitalisation, ICU admission and deaths after infection with SARS-CoV-2 Omicron BA.1 or BA.2 variants compared with Delta for the data set linked to the random sample of whole genome sequenced-SARS-CoV-2 variants, Germany, 1 November 2021 – 15 April 2022 (n=23,257).

Outcome	Delta		Omicron BA.1		Omicron BA.2		OR (95% CI), BA.1 vs Delta	OR (95% CI), BA.2 vs Delta
	n/N	%	n/N	%	n/N	%	Adjusted OR[◊]	Adjusted OR[◊]
**Hospitalisation**	949/ 12694	7.5	226/ 7030	3.2	121/ 3533	3.4	0.30 (0.22 - 0.41) ***	0.22 (0.14 - 0.35) ***
**ICU admission[a]**	189/ 12634	1.5	19/ 7013	0.3	15/ 3525	0.4	0.18 (0.08 - 0.39) ***	0.18 (0.06 - 0.57) **
**Deaths[b]**	229/ 16794	1.4	49/ 9371	0.5	20/ 3624	0.6	0.38 (0.19 - 0.74) **	0.17 (0.07 - 0.41) ***

OR: odds ratio; CI: Confidence interval; NA: not applicable; ICU: intensive care unit

p-value: p<0.05: *, p<0.01: **, p<0.001: ***

[a] the study population contains 85 less cases than that for hospitalisation due to missing values in the variable ICU admission.

[b] Study population from 1 November 2021 – 1 April 2022 (n=29.789)

[◊] OR adjusted for age group, vaccination status, sex, federal state of notifying health authority and week of notification

Figure S2: **Odds Ratio of hospitalisation after infection with SARS-CoV-2 Omicron BA.1 or BA.2 variants compared with Delta according to time after last vaccination, Germany, 1 November 2021 – 15 April 2022 (n=47,160)**

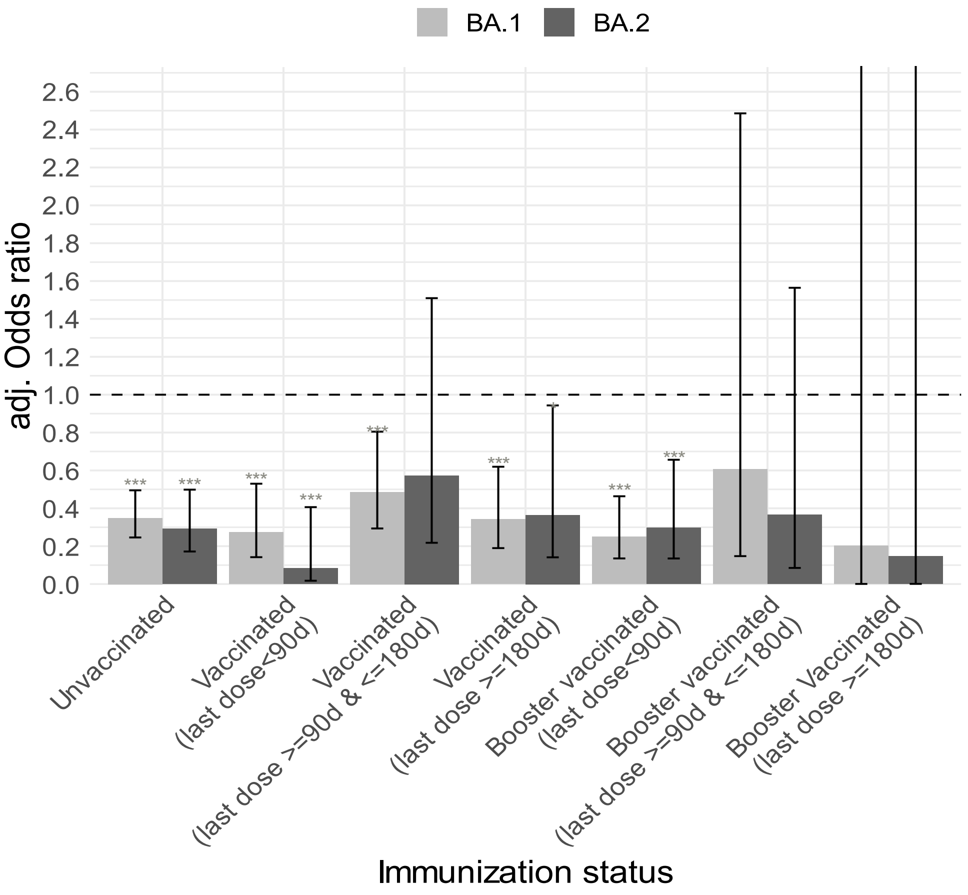

Table S4: Characteristics of COVID-19 cases in the retrospective cohort study on hospitalisation and the overall population, by variants Delta, BA.1 and BA.2 for characteristics variables for severity, Germany, 1 November 2021 – 15 April 2022.

	Study population for hospitalisation (n= 47,160)								Overall population linked with WGS (n= 238,107)							
**Categories**	Delta (B.1.617.2 incl. AY.* lineages)		Omicron -BA.1 (incl. sub-lineages)		Omicron- BA.2 (incl. sub-lineages)		Total		Delta (B.1.617.2 incl. AY.* lineages)		Omicron -BA.1 (incl. sub-lineages)		Omicron- BA.2 (incl. sub-lineages)		Total	
	N	%	N	%	N	%	N	%	N	%	N	%	N	%	N	%
**Total**	24530	52.0	15770	33.4	6860	14.5	47160	100	68875	28.9	96020	40.3	73212	30.7	238107	100
**Sex**																
Female	12401	50.6	8137	51.6	3695	53.9	24233	51.4	34739	50.6	49478	51.8	38382	52.7	122599	51.7
Male	12129	49.4	7633	48.4	3165	46.1	22927	48.6	33872	49.4	46067	48.2	34440	47.3	114379	48.3
**Age group (years)**																
0 - 4	673	2.7	483	3.1	185	2.7	1341	2.8	1602	2.3	2522	2.6	1493	2	5617	2.4
5 - 14	4557	18.6	2255	14.3	792	11.5	7604	16.1	11540	16.8	13087	13.6	7547	10.3	32174	13.5
15 - 34	6190	25.2	5722	36.3	2434	35.5	14346	30.4	18372	26.7	34958	36.4	27578	37.7	80908	34
35 - 59	8807	35.9	5539	35.1	2406	35.1	16752	35.5	25928	37.7	34169	35.6	25822	35.3	85919	36.1
60 - 79	3113	12.7	1238	7.9	728	10.6	5079	10.8	8364	12.1	8009	8.3	7764	10.6	24137	10.1
≥80	1190	4.9	533	3.4	315	4.6	2038	4.3	3059	4.4	3235	3.4	2982	4.1	9276	3.9
**Vaccination status**																
Unvaccinated	14048	57.3	5330	33.8	1897	27.7	21275	45.1	18957	58.3	7830	35.0	2825	28.7	29612	45.8
Vaccinated	9950	40.6	6269	39.8	1554	22.7	17773	37.7	12856	39.6	8756	39.2	2391	24.3	24003	37.1
Booster vaccinated	532	2.2	4171	26.4	3409	49.7	8112	17.2	688	2.1	5768	25.8	4628	47.0	11084	17.1
**Federal state**																
1	4544	18.5	1536	9.7	98	1.4	6178	13.1	21941	31.9	21387	22.3	13859	18.9	57187	24,0
2	5761	23.5	5142	32.6	2589	37.7	13492	28.6	10270	14.9	21258	22.1	20271	27.7	51799	21.8
3	979	4.0	709	4.5	79	1.2	1767	3.7	3284	4.8	4397	4.6	1387	1.9	9068	3.8
4	488	2.0	253	1.6	78	1.1	819	1.7	1670	2.4	1869	1.9	1993	2.7	5532	2.3
5	69	0.3	28	0.2	5	0.1	102	0.2	356	0.5	591	0.6	389	0.5	1336	0.6
6	542	2.2	521	3.3	60	0.9	1123	2.4	1365	2.0	4451	4.6	1837	2.5	7653	3.2
7	1283	5.2	750	4.8	123	1.8	2156	4.6	2581	3.7	4753	5.0	2148	2.9	9482	4,0
8	338	1.4	286	1.8	188	2.7	812	1.7	451	0.7	710	0.7	979	1.3	2140	0.9
9	1044	4.3	609	3.9	276	4	1929	4.1	1827	2.7	2513	2.6	2517	3.4	6857	2.9
10	4962	20.2	3574	22.7	2426	35.4	10962	23.2	11108	16.1	21838	22.7	18890	25.8	51836	21.8

11	1532	6.2	843	5.3	124	1.8	2499	5.3	3046	4.4	4715	4.9	2336	3.2	10097	4.2
12	375	1.5	212	1.3	26	0.4	613	1.3	592	0.9	542	0.6	150	0.2	1284	0.5
13	840	3.4	146	0.9	102	1.5	1088	2.3	6505	9.4	2975	3.1	2949	4	12429	5.2
14	679	2.8	452	2.9	451	6.6	1582	3.4	1983	2.9	1112	1.2	1186	1.6	4281	1.8
15	502	2.0	451	2.9	94	1.4	1047	2.2	680	1	2162	2.3	1551	2.1	4393	1.8
16	592	2.4	258	1.6	141	2.1	991	2.1	1216	1.8	747	0.8	770	1.1	2733	1.1
**Week of notification**																
2021W44	3240	13.2	2	0	0	0	3242	6.9	5608	8.1	4	0	0	0	5612	2.4
2021W45	2710	11.0	4	0	2	0	2716	5.8	5590	8.1	9	0	4	0	5603	2.4
2021W46	2829	11.5	4	0	2	0	2835	6.0	7333	10.6	10	0	2	0	7345	3.1
2021W47	3340	13.6	10	0.1	2	0	3352	7.1	8507	12.4	31	0	5	0	8543	3.6
2021W48	3260	13.3	75	0.5	2	0	3337	7.1	9185	13.3	119	0.1	5	0	9309	3.9
2021W49	3017	12.3	193	1.2	1	0	3211	6.8	8908	12.9	371	0.4	5	0	9284	3.9
2021W50	2715	11.1	558	3.5	1	0	3274	6.9	9131	13.3	1217	1.3	7	0	10355	4.3
2021W51	1408	5.7	918	5.8	4	0.1	2330	4.9	5426	7.9	2165	2.3	10	0	7601	3.2
2021W52	974	4,0	2078	13.2	11	0.2	3063	6.5	4183	6.1	6021	6.3	44	0.1	10248	4.3
2022W1	572	2.3	2367	15.0	35	0.5	2974	6.3	2602	3.8	8339	8.7	180	0.2	11121	4.7
2022W2	283	1.2	2010	12.7	67	1.0	2360	5.0	1332	1.9	9716	10.1	339	0.5	11387	4.8
2022W3	119	0.5	1773	11.2	161	2.3	2053	4.4	503	0.7	10417	10.8	918	1.3	11838	5,0
2022W4	34	0.1	1254	8.0	180	2.6	1468	3.1	245	0.4	10510	10.9	1317	1.8	12072	5.1
2022W5	13	0.1	1022	6.5	203	3.0	1238	2.6	140	0.2	10353	10.8	2032	2.8	12525	5.3
2022W6	7	0	1224	7.8	410	6.0	1641	3.5	73	0.1	10142	10.6	3468	4.7	13683	5.7
2022W7	2	0	811	5.1	477	7.0	1290	2.7	33	0	8360	8.7	4723	6.5	13116	5.5
2022W8	3	0	489	3.1	536	7.8	1028	2.2	26	0	5613	5.8	5523	7.5	11162	4.7
2022W9	3	0	354	2.2	664	9.7	1021	2.2	21	0	4476	4.7	7684	10.5	12181	5.1
2022W10	0	0	325	2.1	931	13.6	1256	2.7	4	0	3524	3.7	9579	13.1	13107	5.5
2022W11	0	0	134	0.8	731	10.7	865	1.8	11	0	2039	2.1	8772	12.0	10822	4.5
2022W12	0	0	78	0.5	591	8.6	669	1.4	7	0	1251	1.3	8237	11.3	9495	4,0
2022W13	1	0	48	0.3	684	10.0	733	1.6	4	0	755	0.8	8130	11.1	8889	3.7
2022W14	0	0	28	0.2	656	9.6	684	1.5	3	0	385	0.4	7368	10.1	7756	3.3
2022W15	0	0	11	0.1	509	7.4	520	1.1	0	0	193	0.2	4860	6.6	5053	2.1

Table S5: Odds Ratio of hospitalisation after infection with SARS-CoV-2 Omicron BA.1 or BA.2 variants compared with Delta, stratified by age group and vaccination status, Germany, 1 November 2021 –15 April 2022 (n=47,160).

Number of hospitalisations and number of cases in group							
	**Age group (years)**	0 - 4	5 - 14	15 - 34	35 - 59	60 - 79	≥80
	**Vaccination status**	n/N	n/N	n/N	n/N	n/N	n/N
Delta	Unvaccinated	36/664	40/4484	122/3494	416/3960	394/1038	267/408
BA.1		21/479	18/2000	34/1300	39/1204	55/242	61/105
BA.2		10/182	3/614	17/478	10/446	19/114	31/63
Delta	Vaccinated	0/9	32/2640	32/2640	134/4666	265/1948	259/616
BA.1		0/2	1/240	29/3132	31/2409	32/386	33/100
BA.2		0/3	1/154	4/752	6/504	12/105	9/36
Delta	Booster vaccinated	0/0	0/2	1/56	6/181	26/127	44/166
BA.1		0/2	0/15	7/1290	16/1926	41/610	54/328
BA.2		0/0	0/24	3/1204	15/1456	24/509	39/216
**Adjusted OR◊**							
	**Age group (years)**	0 - 4	5 - 14	15 - 34	35 - 59	60 - 79	≥80
	**Vaccination status**						
BA.1	Unvaccinated	0.57 (0.23 - 1.42)	0.72 (0.28 - 1.81)	0.46 (0.24 - 0.9) **	0.20 (0.11 - 0.36) ***	0.33 (0.18 - 0.61) ***	0.42 (0.19 - 0.93) *
BA.2		0.73 (0.21 - 2.48)	0.35 (0.05 - 2.35)	0.61 (0.24 - 1.55)	0.12 (0.04 - 0.35) ***	0.20 (0.08 - 0.52) ***	0.37 (0.14 - 0.99) *
BA.1	Vaccinated	NA	NA	0.54 (0.24 - 1.24)	0.30 (0.16 - 0.59) ***	0.42 (0.22 - 0.81) **	0.37 (0.16 - 0.82) **
BA.2		NA	NA	0.28 (0.05 - 1.53)	0.25 (0.06 – 1.00) .	0.44 (0.15 - 1.32)	0.24 (0.06 - 0.91) *
BA.1	Booster vaccinated	NA	NA	0.28 (0.01 - 8.10)	0.19 (0.04 - 0.90) *	0.19 (0.08 - 0.47) ***	0.39 (0.18 - 0.86) **
BA.2		NA	NA	0.13 (0.003 - 5.04)	0.23 (0.05 - 1.12)	0.13 (0.04 - 0.37) ***	0.39 (0.16 - 0.99) *

◊ **OR adjusted for sex, federal state of notifying health authority and week of notification**
